# Pathophysiology of Long Non-coding RNAs in Ischemic Stroke

**DOI:** 10.3389/fnmol.2018.00096

**Published:** 2018-03-29

**Authors:** Weimin Ren, Xiaobo Yang

**Affiliations:** ^1^Center Laboratory, Jinshan Hospital, Fudan University, Shanghai, China; ^2^Department of Neurology, Jinshan Hospital, Fudan University, Shanghai, China

**Keywords:** long non-coding RNA, ischemic stroke, oxygen-glucose deprivation, pathophysiology, diagnostic biomarkers, therapeutic targets

## Abstract

Stroke is a neurological disease with high disability and fatality rates, and ischemic stroke accounts for 75% of all stroke cases. The underlying pathophysiologic processes of ischemic stroke include oxidative stress, toxicity of excitatory amino acids, excess calcium ions, increased apoptosis and inflammation. Long non-coding RNAs (lncRNAs) may participate in the regulation of the pathophysiologic processes of ischemic stroke as indicated by altered expression of lncRNAs in blood samples of acute ischemic stroke patients, animal models of focal cerebral ischemia and oxygen-glucose deprivation (OGD) cell models. Because of the potentially important role, lncRNAs might be useful as biomarkers for the diagnosis, treatment and prognosis of ischemic stroke. This article reviews the functions of lncRNAs in different pathophysiology events of ischemic stroke with a focus on specific lncRNAs that may underlie ischemic stroke pathophysiology and that could therefore serve as potential diagnostic biomarkers and therapeutic targets.

## Introduction

Stroke is an acute cerebral vascular disease with high disability and fatality rate. It is the third most common reason of death in Western countries (Ng et al., 2014; Feigin et al., 2015) and the most common cause of death in China estimated by World Health Organization (Liu et al., 2011; Chen et al., 2017). Each year, approximately 2.4 million people experience a new or recurrent stroke, and 1.1 million people die from stroke. The majority (approximately 75% in China) of acute stroke cases are ischemic stroke (Wang W. et al., 2017). Patients who survive are often burdened with disability and lost productivity, which are socioeconomic burdens (Mozaffarian et al., 2016). As the aging population is increasing in size, the stroke burden in China is likely to increase proportionately.

Ischemic stroke occurs when cerebral blood flow is interrupted, usually due to thrombosis or embolism. Despite decades of research, the treatment for ischemic stroke is limited to thrombolytic therapy and the management of symptoms (dela Peña et al., 2015). The sudden interruption of cerebral blood flow causes cell necrosis and subsequent cerebral edema in the core area of ischemia, followed by the destruction of blood-brain barrier (BBB; Yemisci et al., 2015). The release of necrotic cells induces apoptosis and inflammatory cytokines that cause the death of half of the cells in the vicinity of the infarction and aggravates the brain injury. A variety of complex factors contribute to the destruction of the BBB after ischemic stroke and further aggravate brain injury, including oxidative stress, toxicity of excitatory amino acids, excess calcium ions, increpdased apoptosis and inflammation (Lakhan et al., 2009; Manzanero et al., 2013; Chen et al., 2016; Li G. et al., 2017).

lncRNA is widely involved in basic biological processes such as growth and development, reproductive health and tissue regeneration. However, during the complicated pathophysiological processes of ischemic stroke, the functions and underlying mechanisms of lncRNAs are not fully revealed. lncRNAs, emerging in medical research, are transcripts longer than 200 nucleotides that barely or don’t encode protein (Schaukowitch and Kim, 2014). lncRNAs play crucial roles in regulating the expression level of protein-coding genes and related signaling pathways involved in development of multiple diseases at multiple levels, including epigenetic regulation, transcriptional regulation and post-translational control. As a new type of potential clinical biomarkers with biological functions, lncRNAs have a very wide clinical application prospect. Due to their structural characteristics, lncRNAs can bind homologous genome DNA as well as RNA directly and easily. lncRNAs can also interact with many proteins by forming complex secondary structures. According to the location in genome relative to corresponding coding genes, lncRNAs can be divided into five main types: sense, intronic, antisense, intergenic and bidirectional promoter (Lorenzen et al., 2012; Lorenzen and Thum, 2016). In view of their subcellular localizations, lncRNAs are found to express either in nuclear or cytoplasm. lncRNAs within a cell’s nucleus can recruit chromatin remodeling complexes to specific position on chromosome to induce epigenetic gene silencing. In this way, lncRNAs can regulate specific gene expression locating on the same or another chromosome (Lorenzen et al., 2012). Cytoplasmic lncRNAs act as a molecular sponge against microRNAs to affect microRNAs’ expression and function (Ronco et al., 2008), or target mRNAs directly or indirectly to control transcriptional activity (Yoon et al., 2013).

**Figure 1:**
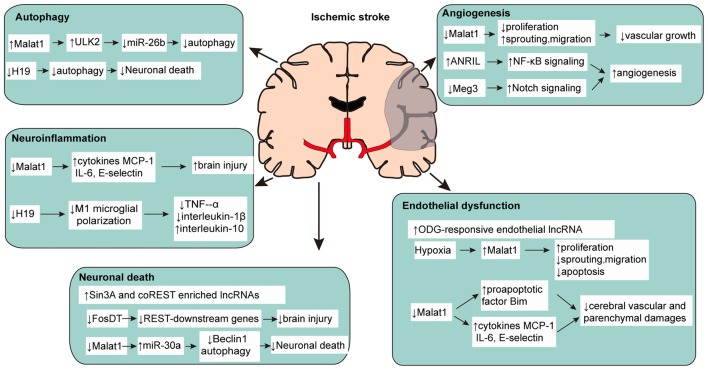
Multiple functions of long non-coding RNAs (lncRNAs) in pathophysiological changes of ischemic stroke.

lncRNAs are among the many classes of molecules that cause functional alterations in ischemic stroke. The study of lncRNAs in ischemic stroke is very limited, but the underlying regulative mechanism cannot be ignored. As an important endogenous regulatory mechanism, lncRNA is expected to become a new modality and target for regulating ischemic stroke (Figure 1, Supplementary Table S1).

Hypertension, diabetes and dyslipidemia are associated with an increased risk for stroke (Howard et al., 2015; Glasser et al., 2016; Kotani et al., 2016). Mazidi et al. (2017) revealed that some lncRNAs are involved in the control of plasma lipid metabolism. For example, ANRIL is likely to be associated with lipid and glucose metabolism by altering the expression of associated genes. LncLSTR may modulate lipid homeostasis by adjusting triglycerides clearance. MALAT1 is associated with diabetes-induced inflammatory response and microvascular dysfunction.

## Aberrant Expression of lncRNAs in Stroke

In contrast to whole-blood RNA samples of healthy adults, altered expression of lncRNAs was found in acute ischemic stroke patients. Dykstra-Aiello et al. (2016) found that the expression levels of 299 lncRNAs in peripheral blood samples of male stroke patients altered, whereas 97 lncRNAs were found in females. Among the above lncRNAs, three (PSR01064543, PSR04026704 and PSR22016723) in males and one (PSR05035106) in female showed differential expression after been filtered statistically on a partial correlation for time since stroke symptom onset (r > |0.4|). Further bioinformatics analysis revealed putatively some of the lncRNAs might be associated with stroke-associated gene like α-Adducins. Aberrant expression of lncRNAs was observed in animal models. Dharap et al. (2012) found that 359 lncRNAs were upregulated and 84 were downregulated among the 8314 lncRNAs in rat cortex subject to transient middle cerebral artery occlusion (MCAO). Of these, 62 lncRNAs showed homology to mRNAs with a >90% sequence similarity. Although they might be considered as pseudogenes, these lncRNAs might stabilize homologous mRNAs to maintain their functions after stroke. Bhattarai et al. (2017) reported that more lncRNAs were observed in ischemic stroke C57BL/6 mice than normal control using RNA-seq, suggesting an active role of lncRNAs in stroke. A cohort of 259 lncRNA were identified and part of them were totally novel. The study demonstrates that ischemia may induce broad alteration of lncRNAs during pathophysiology of stroke in the mouse cortex, which exerts great impact on progression of cerebral stroke.

In addition to the chip research mentioned above, two well studied lncRNAs are reported more often in recent years. H19 is one of the best characterized lncRNA genes and can be activated under hypoxic conditions (Yoshimizu et al., 2008). H19 orthologs in human and mouse show exon structure conservation (Smits et al., 2008). Polymorphisms of the H19 gene have been demonstrated to differentially express and correlate with risk factors for cerebrovascular diseases, such as obesity and high blood pressure (Hernández-Valero et al., 2013; Tragante et al., 2014; Gao et al., 2015; Wang et al., 2017a). Metastasis associated lung adenocarcinoma transcript 1 (MALAT1) is a lncRNA with multiple function. Zhang X. et al. (2017) found that MALAT1, initially correlates with tumor metastasis, increased in cultured cells under oxygen-glucose deprivation (OGD) as well as in mice after MCAO. Both H19 and MALAT1 have complex regulatory mechanisms in ischemic stroke and evidences are emerging.

## Roles of lncRNAs in Post-Stroke Pathophysiology

### lncRNAs and Post-Stroke Neuronal Death

Neuronal damage induced by ischemic stroke can directly result in brain parenchymal damage, thus makes it a potential target for stroke. Evidence shows that lncRNAs participate in this process. Studies have found that neuronal transcriptional repressor element-1 silencing transcription factor (REST) and its corepressors Sin3A and coREST were critical to neuronal death after cerebral ischemia (Noh et al., 2012). Via RIP-chip microarray analysis, Dharap et al. (2013) found 26 of 2497 lncRNAs enriched with Sin3A and 11 of 2497 lncRNAs enriched with coREST significantly increased after stroke in spontaneously hypertensive rats. Since Sin3A and coREST play important roles in post-stroke neuronal death, lncRNAs enriched with Sin3A and coREST show great potential regulatory values in ischemic stroke. For example, lncRNA MRAK159688 enriched with both Sin3A and coREST expressed nearly three times compared to control whereas lncRNA EF094477 enriched with Sin3A increased more than six times after cerebral ischemia. Mehta et al. (2015) reported that focal ischemia also increased binding of the lncRNA Fos downstream transcript (FosDT) to Sin3A and coREST. Furthermore, FosDT knockdown depressed the GRIA2, NFB2, and GRIN1 genes, which are downstream of REST, in the post-ischemic brain. Therefore, FosDT might be an important lncRNA for modulation of ischemic neuronal damage. Guo et al. (2017) found that MALAT1 upregulated in cultured primary cerebral cortex neuron after OGD and downregulation of MALAT1 suppressed neuronal cell death significantly. Further experiment revealed that reduced Beclin-1 mediated autophagy might be one of the mechanisms for the protection of down-regulated MALAT1 on neuron.

### lncRNAs and Ischemia-Induced Cerebral Endothelial Dysfunction

Destruction of BBB is the core event in ischemic stroke and cerebral endothelial dysfunction induced by endothelial injury contributes to exacerbated brain tissue and functional impairment (del Zoppo and Hallenbeck, 2000; Ishikawa et al., 2004; Sandoval and Witt, 2008). More and more evidences suggest that identification of the potential mechanisms and improvement of cerebral endothelial function may effectively reduce brain damage in ischemic stroke (Fagan et al., 2004; Rodríguez-Yíñez et al., 2006; Fisher, 2008). Zhang et al. (2016) identified a total of 147 upregulated and 70 downregulated lncRNA in cultured BMECs after OGD. Snhg12, MALAT1 and lnc-OGD 1006 are among the most upregulated lncRNAs whereas 281008D09Rik, Peg13 and lnc-OGD 3916 are included in the most downregulated lncRNAs as verified by quantitative PCR. Animal experiments demonstrated the similar alteration in mouse ischemic stroke model. The further bioinformatics analysis demonstrated multiple binding sites for transcription factor on lncRNAs induced by OGD, which is a support of transcriptional regulatory of lncRNAs to endothelial dysfunction. Endothelial-selective lncRNAs such as MALAT1 have been demonstrated to have a role in cerebral ischemia and may serve as promising regulators targeting cerebral endothelial dysfunction in ischemic stroke. Michalik et al. (2014) demonstrated that hypoxia induced MALAT1 overexpression promoted human umbilical vein endothelial cells (HUVECs) proliferation, whereas knock-down of MALAT1 inhibited this process significantly and eventually influenced vascular growth. This is similar to the pathological state of cerebral endothelial in ischemic stroke. Xin and Jiang (2017) found that lncRNA MALAT1 overexpressed in cultured primary human cerebral microvascular endothelial cells after OGD and reoxygenation (OGD/R) with activation of PI3K pathway and increased apoptotic rate, suggesting MALAT1 may protect human cerebral endothelial cells injury after OGD/R. Zhang X. et al. (2017) found MALAT1 increased in cerebral microvessels in mice after MCAO as well as cultured mouse BMECs under OGD. Silencing of MALAT1 increased cerebral vascular damage, worsened neurological and motor functions, and induced neuroinflammation in ischemic stroke.

### lncRNAs and Neuroinflammation in Ischemic Stroke

Inflammation occurs in various stages of ischemic stroke and plays a pivotal role. Multiple inflammatory cells and cytokines participate in the process, but the function of lncRNAs on neuroinflammation and ischemic stroke is rarely studied. Early studies have found H19 may suppress tumorigenesis through its long noncoding RNA (Yoshimizu et al., 2008), but recent research reveals the relationship between H19 and neuroinflammation in ischemic stroke. It’s reported that increased H19 levels in stroke patients’ blood impaired neurological function and correlated with tumor necrosis factor-α (TNF-α) levels, which was further verified in animal model (Wang et al., 2017b). Knockdown of H19 by siRNA in MCAO rats reduced brain infarct as well as TNF-α and interleukin-1β (IL-1β), and it increased plasma interleukin-10 (IL-10) concentrations 24 h after stroke. Furthermore, H19 knockdown in BV2 cell-based experiments also reduced TNF-α levels and blocked OGD-driven M1 microglial polarization, which can be turned over by OGD-induced histone deacetylase 1 (HDAC1) overexpression, indicating that H19 facilitates neuroinflammation by promoting HDAC1-dependent M1 microglial polarization. Zhang X. et al. (2017) found that down-regulating MALAT1 significantly increased inflammation related cytokines such as IL-6 and E-selectin in cultured mouse BMECs under OGD. In MALAT1-KO mice, such proinflammatory factors were also found elevated significantly in brain accompanied with larger brain infarct volume and worsened neurological functions. MALAT1 may exerts a protective effect after ischemic stroke via its anti-inflammatory roles in microvasculature.

### lncRNAs and Autophagy in Ischemic Stroke

Current studies show that autophagy is involved in the pathophysiological process of stroke (Qin et al., 2010; Lee et al., 2017). A study found that lncRNA H19 expression was upregulated by cerebral ischemia reperfusion in rats, and by OGD/R *in vitro* in SH-SY5Y cells (Wang et al., 2017a). Inhibition of lncRNA H19 protected cells from OGD/R-induced death by preventing autophagy activation. Furthermore, the authors found that lncRNA H19 inhibited autophagy via the DUSP5-ERK1/2 axis. Li Z. et al. (2017) found that MALAT1 facilitated BMEC autophagy through downregulating miR-26b expression. Through binding to miR-26b, MALAT1 suppressed the effect of miR-26b and further contributed to ULK2 expression. This MALAT1-miR-26b-ULK2 regulatory axis involved in BMEC autophagy provides new insight into the ischemic stroke mechanisms.

### lncRNAs and Angiogenesis in Ischemic Stroke

Angiogenesis may alleviate ischemic necrosis after ischemic injury by assisting brain in restoration of collateral circulation to recover blood supply in damaged parts. Strategies to facilitate angiogenesis are capable of improving the recovery of nervous function and preventing the cognitive deterioration of patients. after stroke. However, very few relevant studies have been conducted. Michalik et al. (2014) confirmed that silencing MALAT1 in HUVECs significantly affects the proliferation capacity and ultimately leads to antiangiogenic effect. Homozygous MALAT1(–/–) mice showed a reduction in vessel density and neovascularization in comparison with wild-type MALAT1(+/+) littermates after *in vivo* angiogenesis was analyzed in neonatal retina model. Subsequent study found that blood flow recovery in hindlimb ischemia mouse model was reduced significantly by pharmacological inhibition of MALAT1, which demonstrated the essential role of MALAT1 in postnatal neovascularization. Zhang B. et al. (2017) found that lncRNA ANRIL significantly increased in rats with diabetes mellitus rats combined with cerebral infarction, and overexpression of ANRIL upregulated and promoted angiogenesis via NF-κB signaling pathway activation. Liu et al. (2017) identified expression of lncRNA Meg3 in rat continuously decreased after MCAO. Further experiments revealed that downregulation of Meg3 improved functional recovery and angiogenesis while overexpression of Meg3 exhibited the opposite effects. These results bring up a hint that low expression of lncRNA Meg3 might be a protective factor for ischemic stroke.

### lncRNAs as Putative Biomarkers and Therapeutic Targets

A series of studies have reported that certain lncRNAs alter differentially over time after ischemic stroke *in vitro* and *in vivo* and could be widely applied in clinic as biomarkers in the future. Observed changes in the levels of lncRNAs in blood samples might be useful biomarkers to reflect the pathophysiological status of the brain, suggesting the promising prospect of circulating lncRNAs. Dykstra-Aiello et al. (2016) also found aberrant lncRNA expression in the peripheral blood of stroke patients with gender difference and suggested that certain lncRNAs might be informative biomarkers for stroke development. Wang W. et al. (2017) reported that variation in the H19 gene increased the risk of ischemic stroke in ischemic patients. Another independent research indicated that the lncRNA H19 levels elevated profoundly in stroke patients’ blood and cell plasma with high diagnostic sensitivity and specificity. Thus suggesting lncRNA H19 could be a new diagnosis or therapeutic target of ischemic stroke. Mehta et al. (2015) suggested that lncRNA FosDT can be therapeutically targeted to reduce brain infarct and post-stroke motor function loss through regulation of genes downstream of REST.

Although we have discovered some functional lncRNAs such as H19 and MALAT1 in ischemic stroke, however, the exploration of lncRNAs still faces multiple challenges. For example, the complexity of lncRNAs’ diverse functions hamper the study of their molecular mechanism, not to mention that many lncRNAs only express in primates. Even if we have confirmed part of the molecular mechanism, there’s still a long way to go before it can be applied to the clinic.

## Conclusion

The understanding of lncRNAs is still at an early stage (Shao and Chen, 2017). Insights into the functions of lncRNAs are thriving in recent 5 years. Although a number of lncRNAs have been found in association with ischemic stroke, the underlying mechanisms are not studied in depth (Boon et al., 2016). Besides, lncRNA cannot be treated separately without insight into other important risk factors of stroke (Glasser et al., 2016). Further studies to reveal more lncRNAs with advancement in bioinformatics and to identify their underlying mechanisms are in urgent need. With advances in our understanding of lncRNAs’ expression and function in the pathogenesis of stroke, we can expect to identify new diagnosis biomarkers and targeted lncRNAs of therapeutic interest in the future. Meanwhile, stroke patients might also benefit from lncRNA-relevant therapy with growing efficacy and accuracy.

## Author Contributions

WR contributed to manuscript writing. XY contributed to figure generation and manuscript writing. All authors read and approved the final manuscript.

## Conflict of Interest Statement

The authors declare that the research was conducted in the absence of any commercial or financial relationships that could be construed as a potential conflict of interest.
