# Supplementary material for: Pathophysiology of Long Non-coding RNAs in Ischemic Stroke
Source: Front Mol Neurosci. 2018 Mar 29;11:96. doi: 10.3389/fnmol.2018.00096 (PMC5884949; doi:10.3389/fnmol.2018.00096)
Supplement: Supplementary file 1 [file Table_1.DOCX]

**Table 1 Altered lncRNAs in ischemic stroke**

| **Tissue/model** | **LncRNA** | **Regulation** | **Pathway targeted by the lncRNAs** | **References** |
| --- | --- | --- | --- | --- |
| whole-blood RNA samples of acute ischemic stroke patients | NR_002332,AJ131606,linc-SLC22A2,linc-OBP2B-1,OTTHUMT00000079682,linc CEP120-1, et al. | up |  | Dykstra-Aiello et al. (2016) |
|  | linc-C10orf57-2, linc-CEP120-1, et al. | down |  |  |
| ipsilateral cortex of spontaneously hypertensive rats, 1 h MCAO and 6 h reperfusion | MRAK077719,XR_009313,XR_007365,MRAK159688,MRAK079854,MRAK049735,MRAK078894,XR_006073,XR_008501,U77626,XR_009083,XR_007044,XR_006491,XR_009527,XR_006222, et al. | up |  | Dharap et al. (2012) |
|  | BC086373,MRAK049859,BC103647,MRAK078348,uc.236+,MRAK040488,MRAK047500,uc.308-,MRAK033792,uc.228+,MRAK051099,MRAK053030,uc.408+,BC158779,S39217 | down |  |  |
| Cortical brain tissue of C57BL/6 mice, MCAO and 6, 12 or 24h reperfusion | MCAO_00130143,MCAO_00080403,MCAO_00037532,MCAO_00043170,MCAO_00082353,MCAO_00123465,MCAO_00123215,MCAO_00051219,MCAO_00035778,MCAO_00130489,MCAO_00035778,MCAO_00017261,MCAO_00030561,MCAO_00123175,MCAO_00080061,MCAO_00009257,MCAO_00140284,MCAO_00074474 | up |  | Bhattarai et al. (2017) |
|  | MCAO_00036742,MCAO_00167460,MCAO_00015087,MCAO_00021315,MCAO_00056214,MCAO_00065334,MCAO_00065346,MCAO_00057347,MCAO_00065461,MCAO_00065454,MC_00065462,MC_00011962,MC_00065509,MC_00176229,MC_00099891,MC_00050563,MC_00054266,MC_00059215,MC_00050564,MCAO_00127304,MCAO_00045496,MCAO_00050562,MCAO_00172799,MCAO_00127300,MCAO_00062531,MCAO_00172798,MCAO_00183738,MCAO_00081134 | down |  |  |
| cortex of spontaneously hypertensive rats, 1 h MCAO and 6 h reperfusion | MRAK154943,XR_005513,MRAK159688,XR_008555,XR_007499,XR_007404,XR_006148,XR_007321,XR_005733,XR_007247,XR_009083,XR_009151,XR_005800,NR_027324,XR_006778,DQ266361,MRAK166199,XR_008295,XR_008876,MRAK049735,XR_007101,XR_007384,AF030089,MRAK135044,AY383714,EF094477,MRAK163011,XR_008508,MRAK143109,XR_008791,MRuc008ymd,MRAK053211,MRAK080604,XR_007355 | up |  | Dharap et al. (2013) |
| tissue from peri-infarct area of spontaneously hypertensive rats, 1h MCAO and 3, 6, and 12 h reperfusion | FosDT | up | FosDT induction and its interactions with REST-associated CMPs, and the resulting regulation of REST-downstream genes might modulate ischemic brain damage. | Mehta et al. (2013) |
| 1)Plasma of ischemic patients  2)BV2 microglial cells subjected to OGD  3)45min MCAO/24 h reperfusion mice | H19 | up | H19 promotes neuroinflammation by driving HDAC1-dependent M1 microglial polarization | Wang et al. (2017) |
| 1)blood samples of ischemic patients  2)SH-SY5Y cells subjected to OGD/R  3)peri-infarct area tissue in 2h MCAO rats | H19 | up | the variation of H19 gene increased the risk of ischemic stroke | Wang et al. (2017) |
|  |  |  | lncRNA H19 inhibited autophagy through DUSP5 -ERK1/2 axis. |  |
| primary BMECs after 16h OGD | Snhg12,Malat1,Srsf3,lnc-OGD1645,Snhg1,Gm11974,lnc-OGD1006,lnc-OGD2327,lnc-OGD 1784,lnc-OGD3838,lnc-OGD2085 | up |  | Zhang et al. (2013) |
|  | 281008D09Rik,Peg13,lnc-OGD3916,C920009B18Rik,Pisd-ps1,lnc-OGD2838,lncOGD301, lncOGD1996, lncOGD4126,lncOGD3114 | down |  |  |
| 1)HUVECs subjected to hypoxia  2)Hindlimb Ischemia Mouse Model  3)Mouse Retinal Angiogenesis Model | Malat1 | up | Silencing of MALAT1 induced a promigratory response and increased basal sprouting and migration, whereas proliferation of endothelial cells was inhibited | Michalik et al. (2014) |
|  |  |  | genetic ablation of Malat1 inhibited proliferation of endothelial cells and reduced vascular growth. |  |
| Primary human brain microvascular endothelial cell OGD/R models | Malat1 | up | Malat1 may protect human brain vascular endothelial cells from OGD-R-induced apoptosis via a PI3K-dependent mechanism | Xin and Jiang (2017) |
| 1）Malat1 knock-out and wild-type mice, 1h MCAO and 24 –72 h reperfusion.  2）BMECs after OGD | Malat1 | up | Malat1 KO mice presented larger brain infarct size, worsened neurological scores, and reduced sensorimotor functions and increased expression of proapoptotic and proinflammatory factors | Zhang et al.(2017) |
|  | Malat1 | up | Silencing of Malat1 aggravated OGD-induced expression of the proapoptotic factor Bim and proinflammatory cytokines MCP-1, IL-7, and E-selectin. |  |
| BMEC after OGD/R | Malat1 | up | Malat1-miR-26b-ULK2 regulatory axis | Li et al. (2017) |
| diabetes mellitus(DM) combined with cerebral infarction(CI) in adult male Wistar rats | ANRIL | up | Overexpressed ANRIL upregulates VEGF and promotes angiogenesis by activating NF-κB signaling pathway in DM + CI rats. | Zhang et al. (2017) |
| 1)MCAO Model in rats  2) HMEC-1 | Meg3 | down | Downregulation of Meg3 expression improves neurobehavioral outcome and promotes angiogenesis both in vitro and in vivo by activating Notch Signaling. | Liu et al. (2017) |

BMECs=Brain microvascular endothelial cells

HMEC-1=Human microvascular endothelial cells - 1

MCAO=middle cerebral artery occlusion

HUVECs=human umbilical vein endothelial cells
